# DIABETES REMISSION RATE IN DIFFERENT BMI GRADES FOLLOWING ROUX-EN-Y GASTRIC BYPASS

**DOI:** 10.1590/0102-672020180001e1343

**Published:** 2018-03-01

**Authors:** Daniel COELHO, Eudes Paiva de GODOY, Igor MARREIROS, Vinicius Fernando da LUZ, Antônio Manuel Gouveia de OLIVEIRA, Josemberg Marins CAMPOS, Silvio da Silva CALDAS-NETO, Mirella Patrícia Cruz de FREITAS

**Affiliations:** 1 Service of Obesity and Related Diseases, University Hospital Onofre Lopes, Federal University of Rio Grande do Norte, Natal, RN; 2 Department of Anesthesiology, Federal University of Rio Grande do Norte, Natal, RN; 3 Department of Pharmacy, Federal University of Rio Grande do Norte, Natal, RN; 4 Federal University of Pernambuco, Recife, PE, Brazil

**Keywords:** Type 2 diabetes mellitus, Gastric bypass surgery, Obesity, Metabolic surgery, Non-severe obesity., Diabete melito tipo 2, Derivação gástrica em Y-deRoux, Obesidade, Cirurgia metabólica, Obesidade não grave.

## Abstract

**Background:**

Type 2 diabetes mellitus has a high long-term remission rate after laparoscopic Roux-en-Y gastric bypass (LRYGB), but few studies have analyzed patients with BMI<35 kg/m^2^.

**Aim:**

To compare glycemic control after LRYGB between BMI 30-35 kg/m^2^ (intervention group or IG) and >35 kg/m^2^ patients (control group or CG) and to evaluate weight loss, comorbidities and surgical morbidity.

**Methods:**

Sixty-six diabetic patients (30 in IG group and 36 in CG group) were submitted to LRYGB. Data collected annually after surgery were analyzed with generalized estimating equations.

**Results:**

Average follow-up was 4.3 years. There was no statistical difference between groups using complete remission American Diabetes Association criteria (OR 2.214, 95%CI 0.800-5.637, p=0.13). There was significant difference between groups using partial remission American Diabetes Association criteria (p=0.002), favouring the CG group (OR 6.392, 95%CI 1.922-21.260). The higher BMI group also had lower HbA1c levels (-0.77%, 95%CI -1.26 to -0.29, p=0.002). There were no significant differences in remission of hypertension, dyslipidemia and surgical morbidity, while weight was better controlled in the IG group.

**Conclusion:**

No differences were found in diabetes complete remission, although greater partial remission and the lower levels of glycated hemoglobin in the BMI >35 kg/m^2^ group suggest a better response among more obese diabetic patients with LRYGB. In addition, both groups had important metabolic modifications at the expense of low morbidity.

## RESUMO

**Racional:**

Diabete mellito tipo 2 apresenta alta taxa de remissão em longo prazo após derivação gástrica em Y-de-Roux (DGYR), mas poucos estudos analisaram pacientes com IMC <35 kg/m^2^.

**Objetivo:**

Comparar o controle glicêmico de pacientes após DGYR entre IMC 30-35 kg/m^2^ (grupo intervenção ou GI) e >35 kg/m^2^ (grupo controle ou GC) e avaliar a perda de peso, comorbidades e morbidade cirúrgica.

**Método:**

Sessenta e seis pacientes diabéticos (30 no grupo GI e 36 no GC) foram submetidos à DGYR. Dados foram coletados anualmente após a operação e analisados com equações de estimativa generalizada.

**Resultados:**

A média de seguimento foi 4,3 anos. Não houve diferença estatística entre os grupos usando critérios de remissão completa da American Diabetes Association (OR 2,214, 95%IC 0,800-5,637, p=0,13). Houve diferença significativa entre os grupos usando critérios de remissão parcial da American Diabetes Association (p=0,002), favorecendo o grupo GC (OR 6,392, 95%IC 1,922-21,260). O grupo com IMC maior também teve menores níveis de HbA1c (-0,77%, 95%IC -1,26 a -0,29, p=0,002). Não houve diferença significativa na remissão de hipertensão, dislipidemia e morbidade cirúrgica, enquanto o peso foi melhor controlado no grupo GI.

**Conclusão:**

Nenhuma diferença foi encontrada na remissão completa do diabete, embora maior remissão parcial e menores níveis de hemoglobina glicada no grupo com IMC >35 kg/m^2^ sugiram melhor resposta entre diabéticos mais obesos com DGYR. Além disso, ambos os grupos tiveram importantes modificações metabólicas às custas da baixa morbidade.

## INTRODUCTION

Improvement or complete remission of type 2 diabetes mellitus (T2DM) has been frequently observed with the advent of surgery for obesity. Complete remission rates of around 71.6% have been reported in studies of patients with obesity grade 2 or 3 after laparoscopic Roux-en-Y gastric bypass (LRYGB)^2^ ^,^ ^24^. Despite the evidence of the effectiveness of bariatric surgery in the treatment of T2DM, its role as a primary therapeutic option for its control in grade 1 obese patients has not yet been defined. The literature regarding this patient population is based on a small number of case series, but results have been encouraging^1^ ^,^ ^8^ ^,^ ^11^ ^,^ ^12^ ^,^ ^15^ ^-^ ^17^ ^,^ ^20^ ^,^ ^22^ ^,^ ^26^ ^,^ ^31^. 

The baseline BMI has been considered a predictive factor of T2DM control post-LRYGB in morbid obesity patients in some publications, but prospective studies including grade I obese patients are scarce^7^ ^,^ ^10^ ^,^ ^11^ ^,^ ^18^ ^,^ ^19^ ^,^ ^30^. A recent meta-analysis did not find a significant difference in remission rates between diabetics with BMI less than 35 kg/m^2^ and BMI of 35 kg/m^2^ or more^28^. There are only two prospective studies comparing LRYGB with differing BMI groups including BMI below 35 kg/m^2^, and no significant differences were found^26^ ^,^ ^29^. Almost all these articles describe Asian patients, so the results may not be the same in Western countries.

The objective of this study was to compare glycemic control by analyzing complete remission rate, partial remission rate and rate of insulin use after LRYGB in grade I obese patients and in grade II or III obese individuals from Brazil and also to analyze weight loss, modification in associated metabolic co-morbidities and surgical morbidity. 

## METHOD

The data was collected prospectively from 66 patients with T2DM who underwent LRYGB from January 2006 to December 2014 retrospectively analyzed. This study was approved by the Research Ethics Committee of Hospital Onofre Lopes, with no requirement for informed consent. Some of these patients had been included in a previous prospective study on LRYGB treatment of T2DM patients with BMI between 30-35 kg/m^2^. Diagnostic criteria of T2DM were according to American Diabetes Association consensus^5^. Patients were included if they were between 18- 65 years-old, both genders, had been submitted to LRYGB surgery and had a diagnosis of T2DM at least two years before surgery. The participants were divided into two groups based on initial body mass index (BMI): lower (BMI <35 kg/m^2^, intervention group or IG), with 30 patients, and higher (BMI >35 kg/m^2^, control group or CG), with 36 patients. In addition, patients with BMI <35 kg/m^2^ were tested for anti-Gad and anti-islet autoimmunity and excluded if any of the tests were positive.

All patients underwent the same technique of LRYGB by a group of three experienced laparoscopic bariatric surgeons. A 20 ml gastric pouch was created using three cartridges of blue 45 mm laparoscopic linear staplers. A 100 cm biliopancreatic limb and a 150 cm antecolic alimentary limb were formed. Two single layer anastomoses were manually made. Patients were encouraged to ambulate early and were fed a liquid diet on the first postoperative day if there were no complications. They were discharged on the second if they mentioned no symptoms and glycemia levels were controlled. Intraoperative and/or postoperative insulin pump was installed according to the capillary blood glucose and changes in infusion were performed according to the evolution. 

The primary outcome was T2DM remission according to the ADA criteria^3^, fasting blood glucose (FBG) <100 mg/dl and hemoglobin A1c (HbA1c) <6.0% without medication. Partial T2DM remission was defined as FBG <125 mg/dl and HbA1c<6.5% without medication. Data were obtained at follow-up visits on anthropometric measures including weight, BMI, total weight loss (TWL), excessive residual weight (ERW) and excessive weight loss (%EWL), and biochemical variables were determined, including FBG, HbA1c, total cholesterol (TC), LDL-cholesterol (LDL), HDL-cholesterol (HDL) and triglycerides (TG). HbA1c was measured by immunoturbidimetry. Both HbA1c and FBG were used to adjust medication. 

### Statistical analysis

Data are expressed as absolute and relative frequencies or mean±SD, as appropriate. Baseline characteristics of groups were compared using Student’s t-test and the Chi-square test or Fisher’s exact test. For the comparison of groups over time, a GEE model with an AR^1^ correlation structure and robust standard errors was used. For the analyses of T2DM remission and partial remission, the binomial family and the logic link were used; for all remaining variables the Gaussian family and the identity link were used. All analyses were adjusted by gender, age at the time of surgery and the baseline value of the dependent variable. For HbA1c and FBG, the analyses were further adjusted by the natural logarithm of the duration of diabetes and by medication use. FBG, TC, HDL, TG, weight, ERW and TWL were log-transformed to obtain normal distributions. Results were presented as point estimates and 95% confidence intervals (CI) of the odds ratio of CG to IG for T2DM remission, of the ratio of CG/IG for log-transformed variables, and of the difference CG-IG for variables in linear scale. Statistical significance was assumed at a two-tailed p-value of less than 0.05. Statistical analysis was performed with the Stata release 11 software (Stata Corporation, College Station, TX, USA).

## RESULTS

Sixty-six patients were included in this study, 30 with a BMI<35 kg/m^2^ and 36 >35 kg/m^2^. Baseline data are shown in Table 1 **.** The groups were fairly similar in all variables except that the group with greater BMI had higher prevalence of arterial hypertension in addition to increased weight, BMI and excessive weight loss. The duration of follow-up was similar between groups: 4.32±1.78 years in IG and 4.34±2.06 years in CG (p=0.97). 

**TABLE 1:** Baseline characteristics of two groups

Variable	IG (n=30)		CG (n=36)		p
Female (n.%)	20	66.7	29	80.6	0.20
Age (m.sd)	47.2	9.8	45.1	9.0	0.36
Pre-operative SAH (n.%)	17	56.7	34	94.4	<0.001
T2DM duration years (m.sd)	8.77	8.48	8.58	9.05	0.94
Pre-operative insulin use (n.%)	16	53.3	15	41.7	0.34
Pre-operative insulin use years (m.sd)	5.39	5.76	8.28	11.43	0.38
Oral hypoglycemic (n. %)	30	100.0	35	97.2	0.36
HbA1c (m.sd)	8.29	1.45	8.52	1.84	0.60
FBG (m.sd)	156.3	59.0	142.2	54.6	0.33
TC (m.sd)	199.2	54.2	200.7	41.7	0.90
HDL (m.sd)	45.1	13.4	45.8	10.5	0.82
LDL (m.sd)	115.1	37.1	110.8	46.3	0.74
TG (m.sd)	227.5	139.6	233.3	135.5	0.88
Weight (m.sd)	86.0	9.75	125.4	26.9	<0.001
BMI (m.sd)	32.3	1.50	49.8	9.80	<0.001

SHA=systemic arterial hypertension; BMI=body mass index; FBG=fasting blood glucose; TC=total cholesterol; LDL=low-density lipoprotein cholesterol; HDL=high-density lipoprotein cholesterol; TG=triglyceride; T2DM=type 2 diabetes mellitus

There was no significant difference between groups in ADA complete remission: the odds ratio of remission in CG vs. IG was 2.214 (95% CI 0.800-5.637, p=0.13). However, the partial remission rate was significantly higher (p=0.002) in CG (O.R 6.392, 95%CI 1.922-21.260). HbA1c levels over time were significantly different between groups, with levels in CG on average 0.77% lower (95% CI -1.26% to -0.29%, p=0.002). There was no difference in the rate of insulin use before and after surgery for both groups (p>0.05). However, the rates of use were lower in the last evaluation in both groups, with levels dropping from around 50% to 13% in IG and 8% in CG.

There was no difference between groups in the other biochemical parameters (Table 2). 

**TABLE 2:** Differences over time between groups in biochemical variables

Variable		n	Difference	IC 95%		p
HbA1c	%	49	-0.769	-1.251	-0.287	0.002
FBG	dif %	52	93.1	83.3	104.2	0.21
TC	dif %	48	95.4	86.1	105.8	0.37
HDL	dif %	37	92.3	84.3	101.0	0.08
LDL	mg/dl	23	-9.04	-28.57	10.48	0.36
TG	dif %	37	93.7	80.0	109.7	0.42

All analyses adjusted gender, age and variable initial evaluation. HbA1c and FBG further adjusted by log T2DM duration and use of medication. Log transformation for FBG, TC, HDL and TG. FBG=fasting blood glucose; TC=total cholesterol; LDL=low-density lipoprotein cholesterol; HDL=high-density lipoprotein cholesterol; TG=triglyceride; dif%=CG average/IG average x 100

There was a statistically significant difference in the excessive weight loss between groups (p>0.05), with total weight loss (TWL) higher in CG (p<0.001, Table 3)**,** but there were no severe nutritional deficiencies in either group. At last evaluation, no patients in IG had obesity while 86.1% of patients in CG continued having some degree of obesity.

**TABLE 3:** Differences over time between groups in anthropometric variables

Variable		n	Difference	IC 95%		p
Weight	dif %	54	103.4	93.1	114.9	0.53
TWL	%	57	20.7	13.1	28.3	<0.001
ERW	dif %	52	106.4	96.0	268.0	0.07
% EWL	%	57	-0.08	-0.20	0.05	0.22
% ERW	%	57	0.08	-0.05	0.20	0.22
Weight variation	kg	57	0.093	0.051	0.136	<0.001
BMI	dif %	54	109.1	96.0	123.9	0.18

All analysis adjusted by gender and age. Weight, ERW and BMI further adjusted by the logarithm of baseline value. Weight, ERW and BMI were log transformed. dif %=average CG/average IG x 100; TWL=total weight loss; ERW=excessive residual weight; EWL=excessive weight loss

There was no mortality in either group. There was no significant difference between the groups related to the main surgical complications (Tables 4 and 5).

**TABLE 4:** Surgical complication rates

Complication	Group BMI <35	Group BMI>35	p
	(n=30)	(n=36)	
Leakage	1	3	0.62
Bleeding	0	0	-
Myocardial infarction	0	1	1
Anastomosis stenosis	1	3	0.62
Sepsis	2	1	0.58
Hypertensive crisis	0	1	1
PE	0	1	1
Total complications	4	10	0.22

BMI=body mass index; PE=pulmonary embolism

**TABLE 5:** Consequences of morbidity

Complication	Group BMI<35	Group BM >35	p
	(n=30)	(n=36)	
ICU admission	4	8	0,54
Reoperation	3	3	1
Readmission	7	11	0.7

BMI=body mass index; ICU=Intensive Care Unit

## DISCUSSION

This study found differences between the groups in T2DM remission according to the partial ADA criteria and no difference in complete remission^3^. Remission rates can vary widely depending on the criteria used. For example, remission rates according to ADA criteria are very different than rates published previously by Buchwald in their meta-analysis^23^. Recent studies have shown lower remission rates when the ADA criteria are used, reaching about 40%, lower than the rates presented in previous studies^5^ ^,^ ^25^. Despite the ongoing discussion about the real usefulness of the ADA remission criteria^27^, they still remain the most popular among studies. This study showed somewhat lower levels of remission in both groups, which can be explained by different follow-up times or severity of disease. Despite the statistically significant difference between groups in HbA1c levels on follow-up, the complete remission criteria did not show difference between groups, possibly because the study was underpowered. Persistent obesity in CG could also explain this difference, by maintaining a greater insulin resistance in this group, “not appearing” in the more stringent complete remission criteria due to underpower, while “being evident” in the partial remission rate and HbA1c differences. Alternatively, a lower partial remission rate and higher glycated hemoglobin levels in the group with lower BMI could be interpreted as evidence of more severe residual disease in patients who did not achieve complete remission. All patients in this group had BMI <30 in the last evaluation, which could lead to adequate control of insulin resistance, so more serious residual disease could be a consequence of worse function of beta cells. Unfortunately, insulin resistance was not measured in this study. This was retrospective, but there are only two comparative prospective studies about this subject: one covered only three patients in the BMI below 35 kg/m^2^ group and the other study found no difference in T2DM remission rates between groups after 36 months of follow-up^26^ ^,^ ^29^. This last study, with a method similar to ours, included 20 patients in the group with BMI <35 kg/m^2^, found a difference in complete remission rates in 12 and 24 months and no difference using partial remission in all follow-up intervals, the exact opposite to these results. However, that difference in complete remission rate was not found at the end of follow-up at 36 months. That study had much higher remission rates in the morbidly obese patients than this one and can explain this difference in results. Another study compared mini gastric bypass between different degrees of BMI and found no statistically significant difference in remission rates. However, there are limitations for traditional bypass comparison by the peculiarities of this surgical technique^21^.

In a recent review, Ngiam KY et al.^24^ used the change in HbA1c as criterion for clinical improvement of patients, commenting on the existing controversy regarding the criteria for remission. That author suggested that the best criteria for the evaluation of surgical effectiveness in T2DM was HbA1c <6.5% and FBG between 100-125 in the absence of medication, that is, the same definition of partial ADA remission. Interestingly, using those criteria we found a statistically significant difference between the groups. Stringent remission criteria may have little clinical practical use. Some patients may potentially benefit from adjuvant medical therapy to achieve optimal metabolic outcomes and this might be considered a good result. Although randomized clinical trials have compared medical and surgical treatment, they are not mutually exclusive, and in practice many patients present good postoperative metabolic control with the use of only one oral hypoglycemic drug and large improvement from the preoperative clinical situation, where there was complete lack of metabolic control. Lakdawala M et al. highlight the importance of avoiding terminology such as postoperative “cure”. Instead, the main result should be the extent of the real benefit of good glycemic control in these patients^15^. 

Most patients in our sample had advanced T2DM, as characterized by long periods of preoperative T2DM, the frequency of insulin use and high preoperative average FBG levels and HbA1c. One of the most frequently cited factors as a predictor of T2DM remission is its preoperative duration^6^. However, our analyses were adjusted to control for differences between groups in all those variables.

Although controversial, postoperative glycemic control has been related to the amount of weight loss in morbidly obese patients. Some studies have found an association^9^ ^,^ ^14^ and others no association^4^ ^,^ ^13^. Lakdawala M et al, studying obese grade I patients, found the weight loss to be a predictive factor of diabetes control in this group of patients^15^. Additionally, Chikunguwo SM found weight regain to be related to T2DM recurrence studying morbidly obese patients^4^. The evidence suggests that weight loss has some role in diminishing peripheral insulin resistance and it can have some implication while comparing groups with diverse initial BMI. Patients with higher BMI lost higher total adipose tissue during the same period, as demonstrated by the different weight loss between the groups. However the proportional weight loss (%EWL) values were similar and it is not known which weight loss is more closely related with peripheral insulin resistance decrease, if absolute or relative. This can be a reason for different response between the groups when hypothetically, CG should have had milder diabetes, “masked” by a higher peripheral insulin resistance, which disappeared after surgery, or higher adipose loss, explaining the better results found in CG. This could be a bias related to comparing similar groups before surgery, but not equal in all aspects. 

We found no difference in the control of hypertension or dyslipidemia between the groups. The same result was reported^21^, but there is scarce information on patients with lower BMI because the focus in these studies is on diabetes remission. We see no reasonable explanation for any difference in the rates of control of dyslipidemia between the groups. However, the absence of difference in hypertension remission rates is possibly due to lack of statistical power, because the BMI >35 kg/m^2^ group had a higher preoperative hypertension rate since surgery should be more effective in control of this co-morbidity in this group than in the BMI <35 kg/m^2^ group.

This study was a retrospective analysis of prospectively collected data based on a small sample and presenting mid-term results. However, it was comparative and included patients operated in the same period by the same group of surgeons. Therefore, despite its limitations, the results are possibly more realistic than non-comparative, short-term results published in most of the previous series. 

Studies of specific subgroups and outcomes more directly related to cardiovascular events and long-term mortality and compared to the best possible clinical treatment are necessary, offering safe identification of the best candidates for surgical treatment. 

## CONCLUSION

There were better results in diabetes control in group with BMI >35 kg/m^2^ comparing with patients with BMI between 30 and 35 kg/m^2^ reflected by better partial remission rate and glycated hemoglobin levels. These differences could be explained by more severe residual post-surgical diabetes. Both groups had significant impact in diabetes control with low morbidity, highlighting the potential of this treatment in patients with lower BMI. 
